# Risk prediction models for dysphagia after radiotherapy among patients with head and neck cancer: a systematic review and meta-analysis

**DOI:** 10.3389/fonc.2025.1502404

**Published:** 2025-02-07

**Authors:** You Pu, Jin Yang, Lian Shui, Qianlong Tang, Xianqin Zhang, Guangguo Liu

**Affiliations:** ^1^ Department of Oncology, Sichuan Mianyang 404 Hospital, Mianyang, Sichuan, China; ^2^ Department of Cardiology, Sichuan Mianyang 404 Hospital, Mianyang, Sichuan, China; ^3^ School of Basic Medical Sciences, Chengdu Medical College, Chengdu, Sichuan, China

**Keywords:** dysphagia, head and neck cancer, radiotherapy, predictive models, meta-analysis

## Abstract

**Background:**

Predictive models can identify patients at risk and thus enable personalized interventions. Despite the increasing number of prediction models used to predict the risk of dysphagia after radiotherapy in patients with head and neck cancer (HNC), there is still uncertainty about the effectiveness of these models in clinical practice and about the quality and applicability of future studies. The aim of this study was to systematically evaluate and analyze all predictive models used to predict dysphagia in patients with HNC after radiotherapy.

**Methods:**

PubMed, Cochrane Library, EMbase and Web of Science databases were searched from database establishment to August 31, 2024. Data from selected studies were extracted using predefined tables and the quality of the predictive modelling studies was assessed using the PROBAST tool. Meta-analysis of the predictive performance of the model was performed using the “metafor” package in R software.

**Results:**

Twenty-five models predicting the risk of dysphagia after radiotherapy in patients with HNC were included, covering a total of 8,024 patients. Common predictors include mean dose to pharyngeal constrictor muscles, treatment setting, and tumor site. Of these models, most were constructed based on logistic regression, while only two studies used machine learning methods. The area under the receiver operating characteristic curve (AUC) reported values for these models ranged from 0.57 to 0.909, with 13 studies having a combined AUC value of 0.78 (95% CI: 0.74-0.81). All studies showed a high risk of bias as assessed by the PROBAST tool.

**Conclusion:**

Most of the published prediction models in this study have good discrimination. However, all studies were considered to have a high risk of bias based on PROBAST assessments. Future studies should focus on large sample size and rigorously designed multicenter external validation to improve the reliability and clinical applicability of prediction models for dysphagia after radiotherapy for HNC.

**Systematic review registration:**

https://www.crd.york.ac.uk/prospero, identifier CRD42024587252.

## 1 Introduction

Head and neck cancer (HNC) is the sixth most common malignancy worldwide, accounting for 4% of cancer incidence (1). Among them, more than 90% of cases are squamous cell carcinoma originating from the upper respiratory and digestive tract, mainly distributed in the oral cavity, larynx and pharynx (2). HNC leads to more than 60,000 deaths per year, with an overall 5-year survival rate of approximately 60% (1). Squamous cell carcinoma is highly sensitive to radiotherapy, so radiotherapy has become the mainstay of treatment for HNC, either alone or in combination with surgery and/or chemotherapy (3). Despite significant advances in radiotherapy techniques, radiotherapy-related toxicity remains an important issue affecting patients’ quality of life and disease prognosis (4).

Dysphagia is one of the common serious complications in HNC patients after radiotherapy, and its incidence is estimated to be about 40% to 50% (5, 6). This symptom not only affects the patient’s eating habits, but may also lead to malnutrition, feeding tube dependence, inhalation airway infections, and emotional problems (4, 7–9). Almost all HNC patients may suffer from varying degrees of dysphagia during radiotherapy as well as in the early and late post-treatment periods, and approximately 50% of patients continue to be affected by this symptom even 6 months after the end of treatment (10). In addition, dysphagia significantly increases the consumption of healthcare resources, and hospitalization costs may increase by up to 40% as a result (11). For young HNC patients, dysphagia after radiotherapy not only poses a challenge to their quality of life, but may also severely affect their ability to return to work and social activities (12).

Therefore, it is essential to identify the high-risk factors for dysphagia after radiotherapy and to develop personalized preventive strategies accordingly. The development of dysphagia is a complex multifactorial interactive process involving multiple predictors such as patient characteristics, treatment modality, tumor stage and radiation dose to the organ at risk (13–16). In recent years, predictive models based on clinical and dosimetric characteristics have shown significant potential in assessing the risk of dysphagia after radiotherapy in patients with HNC (17–19). By analyzing patient information in electronic medical records and incorporating the anatomical distribution of tumors, such models identify groups of patients with similar characteristics, providing strong support for assessing the risk of dysphagia after radiotherapy and making personalized treatment decisions (20, 21).

However, as the number of predictive models increases, these models show significant heterogeneity in terms of methodology, analysis of outcome and applicability. Therefore, it becomes particularly critical to systematically assess the quality and application value of these models. The aim of this study was to provide a reference basis for clinical practice and future research by systematically reviewing and meta-analyzing all published prediction models of post-radiation dysphagia in patients with HNC.

## 2 Methods

The study protocol has been registered in the International Register of Prospective Systematic Reviews (PROSPERO) (registration number: CRD42024587252).

### 2.1 Search strategy

As of August 31, 2024, we searched four databases, PubMed, Cochrane Library, EMbase, and Web of Science, for the following keywords: “Head and Neck Neoplasm”, “Head and Neck Cancer”, “Radiotherapy”, “Radiochemotherapy”, “Pharmacotherapy”, “Targeted Radiotherapy”, “Toxicity”, “Side effect”, “Dysphagia”, “Deglutition disorders”, “Swallowing disorders”, “Tube feed”, “Tube feeding dependency”, “Predictor”, “Model”, “Risk factors”, “Risk score”, “Risk prediction model”. Using PubMed as an example, the detailed search strategy is described in **Supplementary Appendix S1**. In addition, we manually searched the references of studies and reviews to trace other relevant studies. All original predictive modelling studies in English that met the predefined inclusion criteria (PICOTS) were included:

P (population): Patients diagnosed with HNC.
I (index prediction model): All available prognostic models predicting the risk of dysphagia after radiotherapy.
C (comparative model): Not applicable.
O (outcomes to be predicated): Supervisor or objectively diagnosed dysphagia.
T (timing): Outcome measures without any specific limitations within the predictive range.
S (setting): Not limited to any specific clinical setting.

### 2.2 Outcome measures

Results covered dysphagia grades 2 to 4. Evaluation criteria were performed according to “Common toxicity criteria: version 2.0” (22).

Grade 0: none;
Grade 1: mild dysphagia, but can eat regular diet;
Grade 2: dysphagia, requiring predominantly pureed, soft, or liquid diet;
Grade 3: dysphagia, requiring feeding tube, IV hydration or hyperalimentation;
Grade 4: complete obstruction (cannot swallow saliva); ulceration with bleeding not induced by minor trauma or abrasion or perforation.

### 2.3 Inclusion and exclusion criteria

Two searchers independently screened the literature based on the following criteria. Inclusion criteria: a) patients with HNC aged ≥18 years who received radiotherapy; b) construction and/or validation of a predictive model for dysphagia after radiotherapy; c) for repeated studies with the same content, preference was given to studies published more recently or with more comprehensive content. Exclusion criteria: a) case reports, unpublished papers, conference abstracts, or review articles; b) studies for which the full text was not available or the data were incomplete. Two investigators independently screened the literature. Dissenting articles were arbitrated by a third investigator. Basic information and extracted data were collected and cross-checked by the two investigators mentioned above.

### 2.4 Study selection and data extraction

According to the admission criteria, the two investigators first screened the title and abstract of the literature, and then carefully read the full text to confirm whether the criteria were met. According to the checklist for critical appraisal and data extraction for systematic reviews of prediction modelling studies (CHARMS) proposed by Moons et al. (23), a data extraction table was developed. Two investigators independently extracted data including author(s)/year, country, study design, participants, follow-up time, main outcome, incidence of outcomes (%), missing data handling, variable selection, model development method, final predictors, model performance, validation method, model presentation. Any disagreements between authors were resolved by discussion or adjudicated by a third author.

For studies that reported multiple models and clearly indicated the best model, we considered them as reporting a single model and represented the best model. For studies that reported multiple models but did not specify a preferred model, we selected the model with the lowest C-statistic as the most conservative basis for initial assessment. Therefore, each study was treated as reporting only one model.

### 2.5 Quality assessment

In this study, we used prediction model risk of bias assessment tool (PROBAST) (24) to comprehensively assess the risk of bias and applicability of the included prediction models. PROBAST, as a tool specifically designed to assess the risk of bias in predictive model studies, performs a meticulous assessment from four key dimensions: participants, predictors, outcome, analysis, each containing a series of questions to determine the possible risk of bias in studies. In addition, the assessment of applicability is based on the three dimensions of participants, predictors and outcome. The evaluation results are classified according to three levels: “low risk”, “high risk” and “unclear”. In this way, we were able to make a comprehensive judgement on the risk of bias and applicability of each model, and select the best prediction model accordingly. During the evaluation process, two researchers independently evaluated the quality to ensure the objectivity and accuracy of the evaluation. Any disagreements that arose during the evaluation process were resolved through discussion, and a third party was brought in to adjudicate if necessary to reach a consensus.

### 2.6 Statistical analysis

In this study, the area under the receiver operating characteristic curve (AUC) was chosen as an indicator of discrimination ability. AUC was pooled by a random-effects model to assess overall discrimination across all prediction models and across clinical settings. A combined AUC of 0.5 indicates no predictive ability, > 0.5 to ≤ 0.7 indicates weak predictive ability, > 0.7 to ≤ 0.9 indicates excellent predictive ability, and an AUC of 1 is considered a perfect predictive criterion (25). Meta-analysis of the AUC of the model was performed using the “metafor” package in the R software, and inter-study heterogeneity was assessed by the I² statistic, where 25%, 50%, and 75% indicated low, medium, and high heterogeneity, respectively (26). In addition, publication bias was assessed using the Egger’s test, with *p* > 0.05 indicating a low likelihood of publication bias (27).

## 3 Results

### 3.1 Study selection

**Figure 1:**
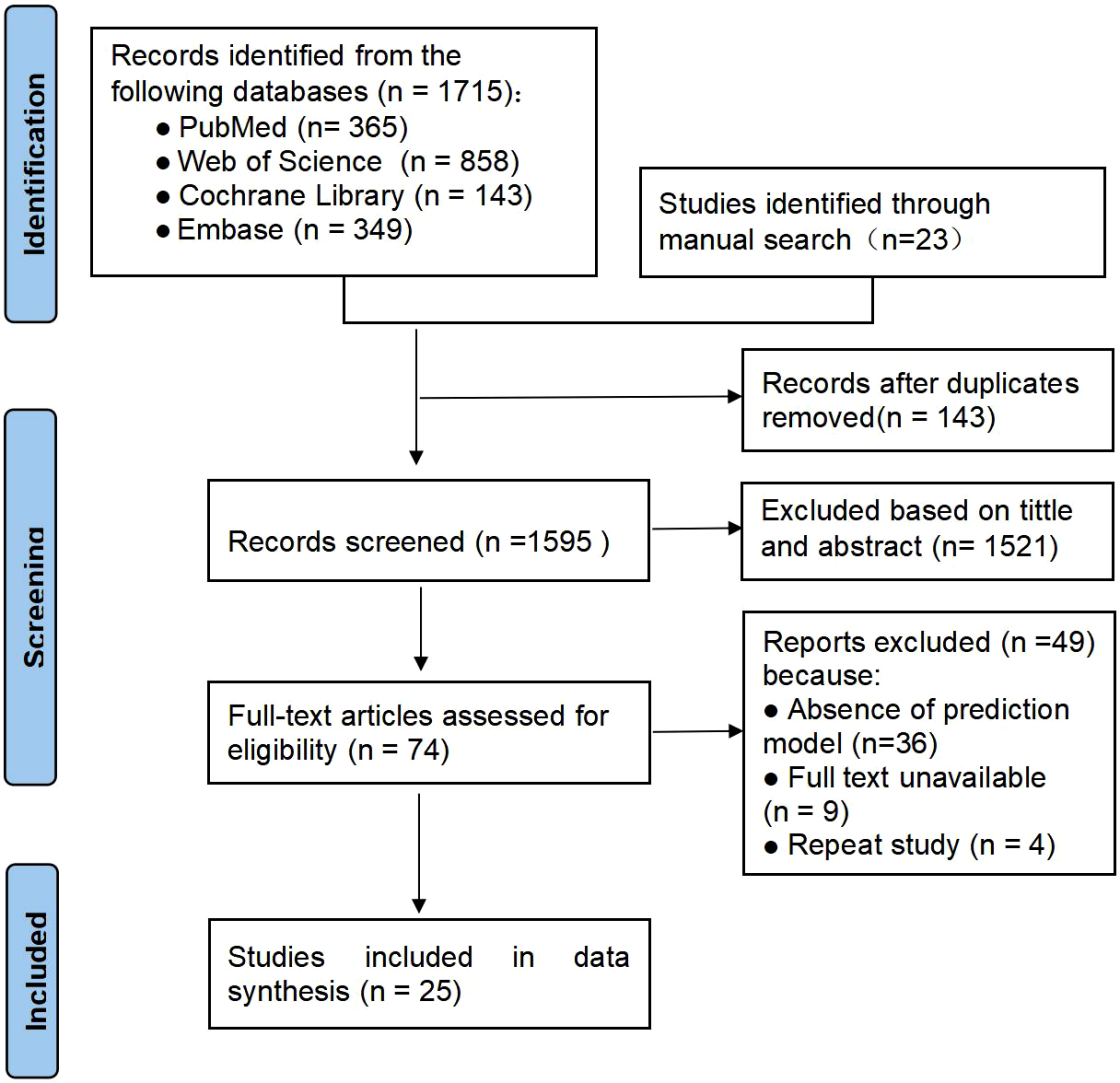
PRISMA flowchart of literature search and selection.

In total 1,715 articles were retrieved through electronic databases and 23 potentially eligible studies were manually retrieved. After deduplication and re-screening, 25 studies involving a total of 8,024 subjects were finally included. The literature screening and selection process is detailed in the PRISMA flowchart in **Figure 1**.

### 3.2 Study characteristics

This study included literature published between 2013 and 2024, mainly from the Netherlands (n = 8), the United States (n = 6), France (n = 2), and the United Kingdom (n = 2). Study types included 10 prospective studies (2 of which were multicenter) and 15 single-center retrospective studies with sample sizes ranging from 23 to 1901 participants. The primary outcome variables studied were dysphagia (n = 17), tube feeding (n = 6), and percutaneous endoscopic gastrostomy insertion (n = 2), with follow-up ranging from 4 weeks to 5 years; and other study details are detailed in **Table 1**.

**Table 1:** Overview of basic data of the included studies.

Author(s), year	Country	Study design	Participants	Follow-up time (after RT)	Main outcome	Incidence of outcomes (%)
Madhavan et al. 2024 (28)	USA	1	HNC patients(231)	3 months	Tube feeding	27.7%
Huynh et al. 2024 (17)	Norway	1	HNC patients(239)	> 5 years	Dysphagia	31.0%
Paetkau et al. 2024 (19)	Canada	1	HNC patients(88)	> 12 months	Dysphagia	A:14%, B:21%
Spiero et al. 2023 (29)	Netherlands	1	HNC patients(1145)	> 6 months	Dysphagia	—
Beddok et al. 2023 (30)	France	1	Recurrent HNC patients(23)	> 3 years	Dysphagia grade ≥2	34.0%
Alexidis et al. 2023 (18)	Greece	1	HNC patients(160)	3 months	Dysphagia grade ≥2	47.5%
Deneuve et al. 2023 (31)	France	2	HNC patients(36)	6 months	Dysphagia grade ≥2	55.5%
Kalendralis et al. 2022 (32)	Netherlands	2	HNC patients(277)	6 months	Dysphagia grade2-4	31.0%
Willemsen et al. 2022 (33)	Netherlands	1	HNC patients(743)	4 weeks	Tube feeding 4 weeks	A: 64%, B: 53%
Gaito et al. 2021 (34)	UK	1	HNC patients(225)	4-6 weeks	Tube feeding>4 weeks	34.7%
Wentzel et al. 2020 (35)	USA	1	HNC patients(200)	6 months	Dysphagia	17.0%
Aylward et al. 2020 (36)	Utah	1	HNC patients(1901)	≥3 years	Dysphagia	8.2%
Karsten et al. 2019 (37)	Netherlands	1	HNC patients(336)	≥3 months	Prolonged (> 90 days) feeding tube dependency	45.0%
Jiang et al. 2018 (38)	China	1	NPC patients(134)	—	Late dysphagia	53.0%
Kanayama et al. 2018 (39)	Japan	1	HNC patients(122)	≥ 6 months	Tube feeding dependence	5.7%
Kamal et al. 2018 (40)	USA	2	OPC patients(97)	3-6 months	Moderate/severe dysphagia	31.0%
Dean et al. 2018 (41)	UK	2multicenter	HNC patients(263)	2 months	Requiring PEG insertion	A: 66%, B: 48%
Alterio et al. 2017 (42)	Italy	2	HNC patients(42)	—	Dysphagia grade ≥ 3/PEG insertion	21.4%
Mavroidis et al. 2017 (43)	USA	2	OPC patients(35)	6 months	Dysphagia	25.7%
Blanchard et al. 2017 (44)	USA	2	HNC patients(192)	6 months	Dysphagia	30.3%
Dale et al. 2016 (45)	USA	1	OPC patients(300)	12 months	Chronic radiation-associated dysphagia	11.0%
Christianen et al. 2016 (46)	Netherlands	2	HNC patients (186)	6 months	Dysphagia grade2-4	22.6%
van der Laan et al. 2015 (47)	Netherlands	2	HNC patients(260)	6 months	Dysphagia grade2-4	24.2%
Wopken et al. 2014 (48)	Netherlands	2multicenter	HNC patients(355)	6 months	Tube feeding dependence	10.7%
Teguh et al. 2013 (49)	Netherlands	1	HNC patients(434)	—	Dysphagia grade>0	66.0%

“—”, not reported; “1”, retrospective study; “2”, prospective cohort study.

HNC, head and neck cancer; NPC, nasopharyngeal cancer; OPC, oropharyngeal cancer; RT, Radiotherapy; PEG, percutaneous endoscopic gastrostomy.

### 3.3 Model construction, performance and presentation

**Tables 2**, **3** summarize the key information for model construction and validation in the studies. For missing value treatment, three studies used different interpolation methods (29, 33, 36), two studies directly deleted missing data (34, 41), and the remaining 20 did not specify the treatment. For predictor screening, one study used univariate analysis (28), one used stepwise logistic regression (40, 47), eight combined univariate analysis and multifactor logistic regression (17, 18, 33, 34, 37, 38, 42, 49), two used Least Absolute Shrinkage and Selection Operator (LASSO) regression (41, 48), and two used recursive partitioning analysis (45), one applied principal component analysis (19), and 10 did not report screening methods. The most common predictors were mean dose to pharyngeal constrictor muscles (PCM) (n=10), treatment setting (n=9) and tumor site (n=7). Other common predictors included age (n=5), tumor stage (n=5), mean dose to oral cavity (n=5), baseline weigh (n=4), baseline dysphagia (n=4), mean dose to the larynx (n=4).

**Table 2:** Construction of the included predictive modelling models.

Author(s), year	Missing data handling	Variable selection	Model development method	Final predictors
Madhavan et al. 2024 (28)	—	Univariate LR	Ridge regression	The dosimetric variables for the DVH metrics model, area deprivation index, baseline weigh, treatment setting, concurrent chemotherapy, bilateral treatment, baseline dysphagia grade 0
Huynh et al. 2024 (17)	—	Univariable analyses, Multivariable LR	LR	Age, female, mean dose to middle PCM
Paetkau et al. 2024 (19)	—	Principal component analysis	Decision tree	The PCM D63% < 55Gy, the superior middle PCM combination structure V31Gy < 100%
Spiero et al. 2023 (29)	Mice imputation	—	Ridge regression	Mean dose to the oral cavity, PCM superior, PCM medius and PCM inferior, dysphagia at baseline, primary tumor location
Beddok et al. 2023 (30)	—	—	LR	Interval to reirradiation, reirradiated volume, mean dose to PCM
Alexidis et al. 2023 (18)	—	Univariate and multivariable LR	LR	The volume in the primary site of disease that received dose ≥ 60Gy, mean dose to the PCM
Deneuve et al. 2023 (31)	—	—	—	Dose to the oral cavity and larynx, volume of PCM
Kalendralis et al. 2022 (32)	—	—	—	Treatment modality, tumor stage, nodal stage, tumor location, the average values of the mean delivered radiation dose
Willemsen et al. 2022 (33)	Stochastic regression imputation	Univariable LR, Multivariable LR	LR	Pretreatment weight change, texture modified diet at baseline, Eastern Cooperative Oncology Group performance status, tumor site, nodal classification, mean dose to the contralateral parotid gland and oral cavity
Gaito et al. 2021 (34)	Removing	Univariable LR, Multivariable LR	LR	Tumor site, tumor stage, chemotherapy drug, mean dose to the contralateral parotid gland
Wentzel et al. 2020 (35)	—	—	LR	Pathological grade, tumor subsites, therapeutic combination, tumor laterality, age, total dose to tumor, spatial features, extended oral cavity predicted dose, mandible predicted dose, medial pterygoid predicted doses, mandible-tumor and medial pharyngeal constrictor-tumor minimum euclidean surface distance
Aylward et al. 2020 (36)	Iterative chained equation imputation	—	Cox regression	Cancer site in the hypopharynx, advanced tumor classification, chemoradiation, preexisting dysphagia, stroke, dementia, esophagitis, esophageal spasm, esophageal stricture, gastroeso-phageal reflux, thrush, chronic obstructive pulmonary disease
Karsten et al. 2019 (37)	—	Univariable LR, Multivariable LR	LR	Pretreatment BMI, weight loss, functional Oral Intake Scale, tumor stage
Jiang et al. 2018 (38)	—	Univariate analysis, Multivariable LR	LR	Mean dose to the superior and inferior constrictor muscles, age
Kanayama et al. 2018 (39)	—	—	—	Mean dose to the supraglottic larynx, contralateral parotid gland and oral tongue
Kamal et al. 2018 (40)	—	LR (Stepwise regression)	LR	Treatment modalities, tumor category, radiotherapy dose, baseline dysphagia grade, dose to the superior PCM V55 and geniohyoid muscle V69
Dean et al. 2018 (41)	Removing	LASSO	Penalized LR, SVM, RF	Age, male, primary disease site, radiotherapy technique, radiotherapy dose fractionation, concurrent chemotherapy
Alterio et al. 2017 (42)	—	Univariable LR, Multivariable LR	LR	Cervical esophagus V45, cricopharyngeal muscle Dmean
Mavroidis et al. 2017 (43)	—	—	—	The dose/volume metrics of the superior PCM
Blanchard et al. 2017 (44)	—	—	—	Dmean superior PCM, Dmean Supraglottic Larynx
Dale et al. 2016 (45)	—	Recursive partitioning analysis, Multivariable LR	LR	Mylo/geniohyoid complex V69, age
Christianen et al. 2016 (46)	—	—	—	Dose to the superior PCM and supraglottic larynx
van der Laan et al. 2015 (47)	—	Multivariable LR (Stepwise regression)	LR	Acute dysphagia and acute xerostomia in weeks 3–6 of radiotherapy
Wopken et al. 2014 (48)	—	Univariable analysis, LASSO	LASSO analysis	Tumor stage, moderate to severe weight loss at baseline, treatment modalities, mean dose to the superior and inferior PCM, contralateral parotid gland and cricopharyngeal muscle
Teguh et al. 2013 (49)	—	Univariable LR, Multivariable LR	LR	Age, bilateral/unilateral neck irradiation, dose, tumor stage, tumor site

“—”, not reported.

LR, logistic regression; DVH, Dose-Volume Histogram; PCM, pharyngeal constrictor muscles; BMI, body mass index; Dmean, mean dose; LASSO, Least Absolute Shrinkage and Selection Operator; SVM, support vector machine; RF, random forest.

**Table 3:** Performance and presentation of the included predictive model models.

Author(s), year	Model performance		Validation method		Model presentation
	Discrimination	Calibration method	Internal	External	
Madhavan et al. 2024 (28)	AUC=0.87(0.05)	—	5-fold cross-validation	—	—
Huynh et al. 2024 (17)	AUC=0.72	Brier Score, Cal plots	Bootstrapping	—	—
Paetkau et al. 2024 (19)	A: accuracy=73 ± 7%, sensitivity=100 ± 0%; B: accuracy=79 ± 8%, sensitivity =81 ± 20%	—	Random splitting (8:2)	Time validation	—
Spiero et al. 2023 (29)	AUC=0.74	Cal curve	—	Spatial validation	—
Beddok et al. 2023 (30)	AUC= 0.78 (0.53-1)	HL test	Bootstrapping	—	—
Alexidis et al. 2023 (18)	C-statistic = 0.835	HL test	—	—	—
Deneuve et al. 2023 (31)	AUC=0.57(0.40-0.74)	—		External validation*	—
Kalendralis et al. 2022 (32)	AUC=0.83(0.78-0.88)	HL test, Brier scores	—	External validation*	—
Willemsen et al. 2022 (33)	A: AUC=0.728, B: AUC=0.624	HL test	—	Spatial validation	Formula
Gaito et al. 2021 (34)	AUC=0.745(0.678-0.812)	HL test	Random splitting (8:2)	—	Formula
Wentzel et al. 2020 (35)	AUC=0.84	—	Leave-one-out cross-validation	—	—
Aylward et al. 2020 (36)	AUC=0.7271(5 years) AUC=0.7195(10 years) AUC=0.7542(15 years)	—	Random splitting (7:3)	—	—
Karsten et al. 2019 (37)	AUC=0.69	HL test	Bootstrapping	—	Nomogram
Jiang et al. 2018 (38)	AUC=0.726(0.632–0.821)	—	—	—	—
Kanayama et al. 2018 (39)	AUC = 0.79 (0.65-0.90)	HL test, Cal plot	—	External validation*	—
Kamal et al. 2018 (40)	AUC = 0.909	—	Random splitting (8:2)	—	—
Dean et al. 2018 (41)	A: AUC=0.76 (0.08) B: AUC=0.82 (0.04)	Brier score, Cal curve	Random splitting (8:2) +cross-validation	Spatial validation	—
Alterio et al. 2017 (42)	AUC = 0.82(0.69-0.95)	Cal plots	Bootstrapping	—	—
Mavroidis et al. 2017 (43)	AUC = 0.74	—	—	External validation*	—
Blanchard et al. 2017 (44)	AUC = 0.708(0.59–0.82)	HL test	_	External validation*	—
Dale et al. 2016 (45)	AUC=0.835	—	Cross validation	—	—
Christianen et al. 2016 (46)	AUC=0.75 (0.68-0.82)	HL test, Brier scores, Cal plots	_	External validation*	—
van der Laan et al. 2015 (47)	AUC=0.849 (0.797-0.901)	HL test	Bootstrapping	—	—
Wopken et al. 2014 (48)	AUC=0.88	HL test, Cal plot	10-fold cross-validation	—	Formula
Teguh et al. 2013 (49)	AUC=0.712 (0.655-0.768)	—	—	—	Nomogram

“—”, not reported; “*”, The study only involves the validation of the model; “A”, development cohort; “B”, validation cohort.

AUC, area under the curve; HL, Hosmer-Lemeshow; Cal, Calibration.

In most studies, logistic regression is the preferred method for constructing the model, but some studies have used cox regression (36), ridge regression (28, 29), LASSO regression (48), and decision tree (19), support vector machine (41), random forest (41), and other machine learning methods. Specifically, 19 studies focused on model development and validation, while six studies performed only model validation (31, 32, 39, 43, 44, 46). During model validation, 12 studies explicitly reported internal validation methods (17, 28, 30, 34–37, 40, 42, 45, 47, 48), four studies conducted internal and external validation (19, 29, 33, 41), six studies conducted external validation only (31, 32, 39, 43, 44, 46), and three studies did not specify their validation methods. In the process of internal validation, bootstrapping methods were used in five (17, 30, 37, 42, 47), cross-validation in four (28, 35, 45, 48), while three studies were performed by random sample splitting (34, 36, 40). In terms of model presentation, only five studies were presented, three of which were calculated using formulas (33, 34, 48) and two in the form of nomograms (37, 49).

In terms of model performance assessment, discrimination is a widely reported metric. In 25 studies, the values of AUC or C-index ranged from 0.57 to 0.909. In terms of model calibration, 15 models were evaluated, of which 11 studies used the Hosmer-Lemeshow test to assess the calibration of models (18, 30, 32–34, 37, 39, 44, 46–48). In addition, seven studies visualized the model calibration by drawing calibration plots (17, 29, 39, 41, 42, 46, 48), while four studies reported Brier scores to measure the predictive accuracy of the model (17, 32, 41, 46). Notably, only one study assessed the clinical utility of the model (37).

### 3.4 Results of quality assessment

Several problems in the assessment of risk of model bias were identified through the qualitative analyses conducted using the PROBAST tool. In the domain of participants, the main problem lies in the inappropriateness of data sources, such as excessive reliance on data from retrospective studies (17–19, 28–30, 33–39, 45, 49). In the domain of predictors, some studies failed to report quality control measures for predictors (19, 34, 38, 39, 42, 44, 49) and all studies did not explicitly state whether blinding was used in assessing predictor variables. In the domain of outcome, there were problems with non-standardized definitions of endpoints (19, 38, 39, 43–45); and failure to exclude factors that overlap with predictors (29, 30, 32, 35, 36, 40, 45). In addition, all models lacked information on the blinded assessment of outcome-predictor relationships and failed to clarify whether there was an appropriate time interval between predictor assessment and outcome determination. In the domain of analysis, there were insufficient sample sizes, failure to meet the recommendation of “events per variable” (EPV) of more than 10 (17, 19, 30, 31, 35, 38–43), improper handling of missing data (34, 41), and reliance on univariate analysis for variable selection (17, 18, 28, 33, 34, 37, 38, 42, 49). Meanwhile, model calibration was not assessed (19, 28, 31, 35, 36, 38, 40, 43, 45, 49). In terms of internal validation of the models, three studies relied only on a single randomized split sample (34, 36, 40) and none of the studies provided information on data complexity. The applicability risk assessment showed that most of the studies were rated as high risk. Overall, all studies in this systematic review showed a high risk of bias, suggesting that there may be methodological problems during the development or validation of the models (See **Table 4**).

**Table 4:** PROBAST results of the included studies.

Author(s), year	ROB				Applicability			Overall	
	Participants	Predictors	Outcome	Analysis	Participants	Predictors	Outcome	ROB	Applicability
Madhavan et al. 2024 (28)	+	?	+	+	−	−	+	+	+
Huynh et al. 2024 (17)	+	?	+	+	−	?	−	+	?
Paetkau et al. 2024 (19)	+	+	+	+	−	?	+	+	+
Spiero et al. 2023 (29)	?	?	+	?	−	?	+	+	+
Beddok et al. 2023 (30)	+	+	+	+	−	?	+	+	+
Alexidis et al. 2023 (18)	+	?	+	+	−	−	+	+	+
Deneuve et al. 2023 (31)	?	?	+	+	−	?	+	+	+
Kalendralis et al. 2022 (32)	?	?	+	+	−	?	−	+	?
Willemsen et al. 2022 (33)	+	?	+	+	−	?	−	+	?
Gaito et al. 2021 (34)	+	+	+	+	−	−	+	+	+
Wentzel et al. 2020 (35)	+	−	+	+	−	−	+	+	+
Aylward et al. 2020 (36)	−	?	+	+	−	?	+	+	+
Karsten et al. 2019 (37)	+	?	+	+	−	−	−	+	−
Jiang et al. 2018 (38)	+	+	+	+	−	?	+	+	+
Kanayama et al. 2018 (39)	+	+	+	+	−	?	+	+	+
Kamal et al. 2018 (40)	−	?	+	+	−	?	−	+	?
Dean et al. 2018 (41)	−	?	+	+	−	?	+	+	+
Alterio et al. 2017 (42)	−	+	+	+	−	?	+	+	+
Mavroidis et al. 2017 (43)	+	+	+	+	−	?	+	+	+
Blanchard et al. 2017 (44)	−	+	+	+	−	?	+	+	+
Dale et al. 2016 (45)	+	?	+	+	−	?	+	+	+
Christianen et al. 2016 (46)	+	?	+	+	−	?	−	+	?
van der Laan et al. 2015 (47)	−	−	+	+	−	?	−	+	?
Wopken et al. 2014 (48)	−	?	+	+	−	?	−	+	?
Teguh et al. 2013 (49)	+	+	+	+	−	?	+	+	+

“+”, high risk of bias/high concern of applicability; “−”, low risk of bias/low concern of applicability; “?”, no information.

PROBAST, prediction model risk of bias assessment tool; ROB, risk of bias.

### 3.5 Meta-analysis results

**Figure 2:**
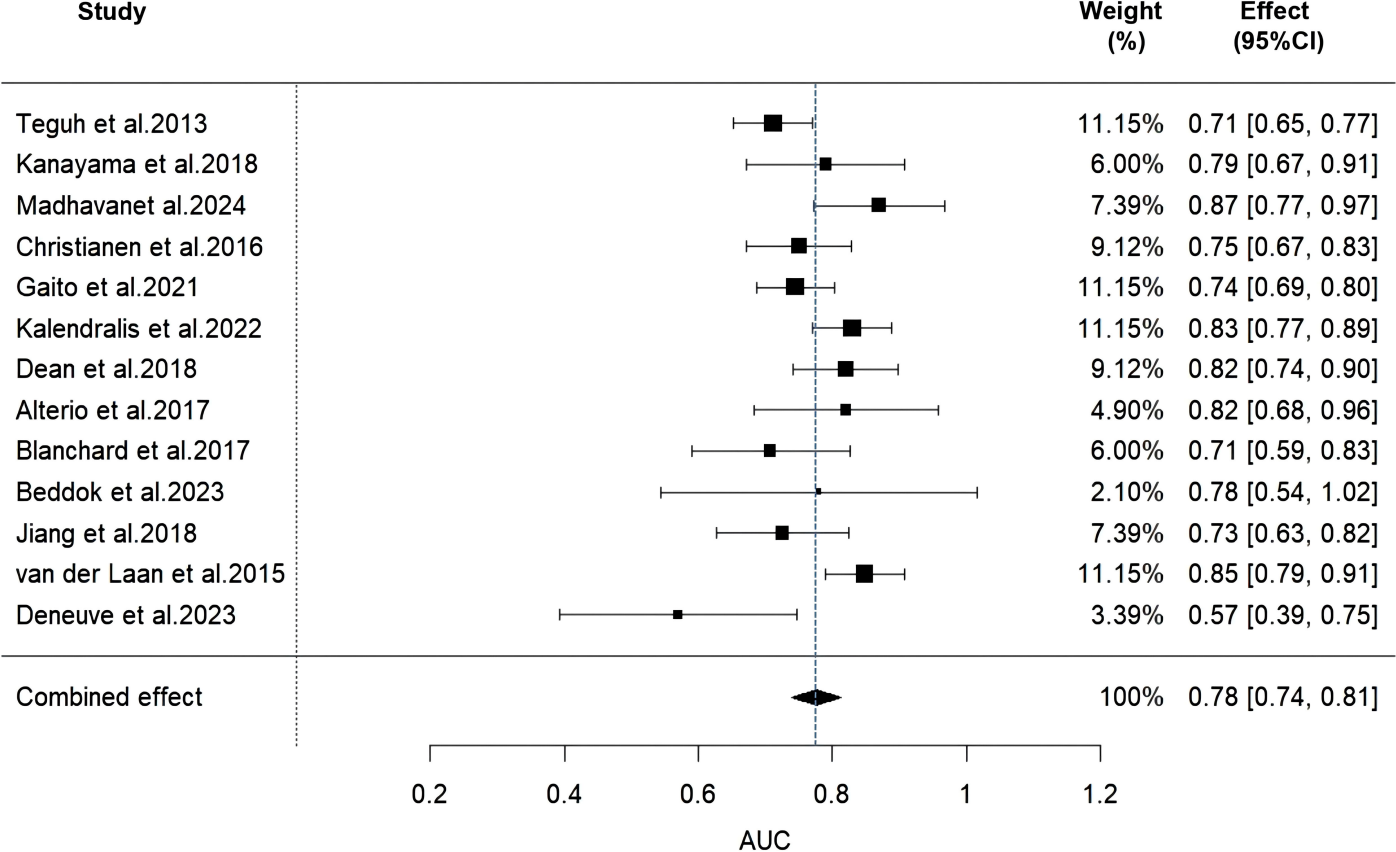
Forest plot of the random effects meta-analysis of combined AUC estimates for 13 validation models.

Due to insufficient details reported by the models of some included studies, only 13 studies were ultimately eligible and included in the Meta-analysis. We used a random-effects model to calculate the combined AUC value, which yielded a result of 0.78 (95% CI: 0.74-0.81) (**Figure 2**). The I² value was 55.23% and the p-value was less than 0.01, which indicated a moderate degree of heterogeneity between studies. Furthermore, the Egger’s test showed a z-value of -0.984 (*p* = 0.325), indicating that there was no statistical publication bias.

## 4 Discussion

In this study, we systematically reviewed 25 predictive models of dysphagia after radiotherapy for HNC, which demonstrated moderate to good predictive performance in internal or external validation, with AUC values ranging from 0.57 to 0.909. However, according to the PROBAST checklist, all included studies were rated as having a high risk of bias, which limited the application of the model in clinical practice. In addition, in a Meta-analysis of 13 validated models, we found that the combined AUC value was 0.78 (95% CI: 0.74-0.81), a result that implies that there is still room for improvement in the discriminatory power of these models. In addition, there was significant heterogeneity among the models, with an I² value of 55.23% and a p-value of less than 0.01, which may be attributed to differences in study design, sample size, predictor selection, and outcome definition.

In this study, we conducted an in-depth analysis of the risk of bias in prediction models. Two of the studies (41, 48) achieved high AUC values (0.82 and 0.88, respectively) through a multicenter prospective cohort design, which to some extent predicts the potential for clinical application. However, the study by Dean et al. (41) was limited by a small sample size (n=263) and the direct exclusion of missing data in data processing, which may have introduced information loss and selectivity bias (50). The study by Wopken et al. (48) faced a similar sample size issue (n=355) and lacked external validation, which further limited the generalizability of their model. In contrast, the two studies with sample sizes of more than 1000 cases (29, 36) were retrospective studies, in which Aylward et al. (36) performed internal validation of their model through a random sample splitting method. However, this method may be affected by chance factors, which may bias the assessment of model performance. Especially in the case of small sample sizes, this random splitting may further weaken the generalization ability of the model. In all the predictive models included in this study, the sample sizes ranged from 23 to 1901 participants, and most studies failed to meet the recommended criterion of at least 10 events (EPV≥10) per predictor variable (51), which may weaken the prediction accuracy of the models. Generally speaking, a larger sample size helps to improve the reliability and stability of the model. Therefore, future research should focus on expanding the sample size to improve the clinical application value of the model.

In this study, we paid particular attention to the transparency and reproducibility of the research methodology. We noted that none of the included studies explicitly reported whether the assessment of outcome measures and predictors was blinded. Lack of blinding may expose assessors to subjective bias, which in turn may affect the objectivity and reliability of the study results. Furthermore, we found that some studies relied on univariate analysis to screen variables, an approach that may fail to adequately consider the interactions between variables, thereby increasing the risk of model bias and potentially leading to the omission of important predictor variables. In order to improve the stability and predictive ability of the model, we suggest adopting more advanced variable screening methods, such as LASSO regression (52), which can deal with the multicollinearity problem among variables and help to identify the most predictive variables. In this systematic review, Paetkau et al. (19) and Dean et al. (41) used machine learning algorithms in model development. Although machine learning algorithms have the potential to improve prediction accuracy (53), they did not demonstrate significant benefits in this review. We believe that this phenomenon may be related to factors such as insufficient sample size, variable screening methods based on univariate analysis, and random division of data sets.

In addition to discrimination, calibration is also a key indicator when evaluating clinical prediction models. Calibration reflects the agreement between the predicted probability of a model and the actual observed probability, which is usually assessed through calibration plots (54). In this study, we found that 15 out of 25 models were assessed for calibration using the Hosmer-Lemeshow test, calibration plots, and Brier scores. However, 10 models did not report calibration results, and although most of these models showed good discrimination with AUC values greater than 0.7, the lack of calibration data may increase the risk of model bias and limit a comprehensive assessment of model performance. Therefore, future studies recommend comprehensive reporting of model results to help clinical staff assess model performance more comprehensively thus better support clinical decision-making.

In this review, we identified and evaluated a series of clinically significant predictor variables that are critical for predicting dysphagia after radiotherapy in patients with HNC. Studies have shown that the average radiotherapy dose to the PCM is a key predictor of the risk of dysphagia after radiotherapy (18, 30). An in-depth study of the effect of dose limitation of the local PCM and its combined substructures on radiosensitivity further revealed the important role of these regions in risk prediction (29, 55) and validated the applicability of the normal tissue complication probability (NTCP) model. The NTCP model emphasizes the key role of dose limitation in PCM and supraglottic laryngeal region in predicting dysphagia after radiotherapy, which provides an important reference for clinical radiotherapy strategies (46). Furthermore, treatment modalities, particularly the combination of radiotherapy and chemotherapy, have been shown to significantly increase the risk of dysphagia (56–58). This increased risk may stem from the synergistic effect between radiotherapy and chemotherapy, exacerbating tissue damage, including mucositis, fibrosis and atrophy, further deteriorating swallowing function (59). The location of the tumor has also been found to be a key predictor of postoperative dysphagia, with different sites of tumors having varying effects on the function of surrounding tissues and organs, especially those structures directly involved in the swallowing process (60). The effect of age as another important predictor on the risk of dysphagia is associated with radiotherapy-induced tissue fibrosis and atrophy (59). This risk rises significantly with age, which may be related to increased comorbidities and decreased body reserve capacity in older patients (61). Among all clinical variables, T stage of the tumor (especially T4 stage) is the strongest predictor of dysphagia because it not only reflects the size of the tumor, but also reveals the aggressiveness and spread extent of the tumor, which together influence the choice and intensity of treatment options (62). Finally, the mean dose to oral cavity was also shown to be an important predictor of dysphagia after radiotherapy. Radiotherapy may lead to reduced elasticity and contraction ability of oral and pharyngeal muscles, affecting normal swallowing function, and may reduce the sensitivity of swallowing reflex, increasing the risk of choking or swallowing by mistake when eating (63).

Therefore, identifying and understanding these predictors is essential for optimizing clinical treatment strategies. When designing and improving predictive models, these key factors should be prioritized for incorporation to increase the value of the models in guiding clinical decisions. Especially in radiotherapy, dose-volume limitation has become an important clinical consideration. For example, limiting the radiation dose for PCM and supraglottic larynx not only effectively reduces the incidence of dysphagia, but also provides key parameters for optimizing prediction models. Future studies should further explore the applicability and impact of these factors and their dose-volume limitations in different patient populations, so as to enhance the clinical relevance and accuracy of prediction models and provide more reliable support for individualized treatment strategies.

This systematic review has the following limitations: (1) This study included only the literature published in English on prediction models for dysphagia risk, which may have led to our failure to cover important research findings in other languages. (2) Due to the heterogeneity of the included studies, we were only able to perform a meta-analysis of some of the studies, which limited our in-depth analysis of the sources of heterogeneity and potential publication bias. (3) Most models have not yet included some commonly used variables, such as xerostomia and severe acute toxicity (mucositis). It is recommended that future studies fully consider the inclusion of these recognized predictors into the model to further improve the accuracy and clinical applicability of the prediction. (4) The high variability observed in the study may be partly due to differences in methodology and treatment options (such as the definition of dysphagia grading, chemotherapy standards and irradiation techniques, etc.). Due to the lack of unified standards, further subgroup analysis cannot be carried out, thus limiting our accurate identification of specific sources of heterogeneity in predictive model research. In order to improve the clinical applicability and scientific accuracy of predictive models, future studies should consider these limitations and take appropriate improvements during study design and execution.

## 5 Conclusion

In this systematic review, we comprehensively analyzed 25 models predicting the risk of dysphagia after chemoradiotherapy in patients with HNC. Although some models showed good predictive performance, all included studies were assessed as having a high risk of bias in methodological quality, which limits the potential use of these models in clinical practice for prophylactic treatment of people at high risk of dysphagia after radiotherapy. In order to improve the quality of future studies and the clinical applicability of the models, investigators should strictly follow methodological and reporting guidelines and systematically evaluate the model development and validation process to reduce the risk of bias. In addition, conducting more external validation studies is essential to comprehensively assess the performance of existing models, which will help to guide clinical decision-making and practice more effectively.

## Data Availability

The original contributions presented in the study are included in the article/**Supplementary Material**. Further inquiries can be directed to the corresponding authors.
