# Supplementary material for: Risk prediction models for dysphagia after radiotherapy among patients with head and neck cancer: a systematic review and meta-analysis
Source: Front Oncol. 2025 Feb 7;15:1502404. doi: 10.3389/fonc.2025.1502404 (PMC11842330; doi:10.3389/fonc.2025.1502404)
Supplement: Supplementary file 1 [file Table1.docx]

Supplementary Material

# Supplementary Tables

| **Appendix S1** PubMed searchable. | |
| --- | --- |
| Number | Search terms |
| #1 | ("Head and Neck Neoplasms" [MeSH Terms]) OR (“Head and Neck Neoplasm*” [Title/Abstract]) OR (“Head and Neck Cancer” [Title/Abstract]) |
| #2 | (Radiotherapy [MeSH Terms]) OR (Radiotherapy [Title/Abstract]) OR (radiochemotherapy [Title/Abstract]) OR (pharmacotherapy [Title/Abstract]) OR ("Targeted Radiotherapy"[Title/Abstract]) |
| #3 | ("Deglutition Disorders"[MeSH Terms]) OR (toxicit* [Title/Abstract]) OR ("side effect*"[Title/Abstract]) OR (dysphagia [Title/Abstract]) OR ("deglutition disorders"[Title/Abstract])) OR ("swallowing disorders" [Title/Abstract])) OR ("tube feed*" [Title/Abstract]) |
| #4 | (predict* [Title/Abstract]) OR (model* [Title/Abstract]) OR ("risk factors" [Title/Abstract]) OR ("Risk Score" [Title/Abstract]) OR ("Risk prediction model" [Title/Abstract]) |
| #5 | #1 AND #2 AND #3 AND #4 |
